# Postoperative continuous compression bandaging was a useful technique for improving pseudosyndactyly in recessive dystrophic epidermolysis bullosa patients

**DOI:** 10.1111/1346-8138.17509

**Published:** 2024-10-16

**Authors:** Kosuke Mochida, Yukiyo Narita, Masahiro Amano

**Affiliations:** ^1^ Department of Dermatology, Faculty of Medicine University of Miyazaki Miyazaki Japan

Dear Editor,

Recessive dystrophic epidermolysis bullosa (RDEB) is a genetic skin disorder characterized by variants in the *COL7A1* gene, which encodes type VII collagen, essential for the basement membrane zone and the formation of anchoring fibrils. Because of the reduced or absent type VII collagen, blistering and subsequent scarring of the hands and feet lead to fusion of the digits accompanied by contractures and pseudosyndactyly. Repeated surgical intervention is often necessary to temporarily improve hand function and delay the recurrence of deformity.^1^

A 36‐year‐old woman with RDEB was referred to our department for pseudosyndactyly of the left hand, having undergone four surgeries previously, the latest 3 years earlier (Figure 1a,b). Owing to insufficient donor sites for skin grafts, artificial dermis was used for wound coverage. The operation began with removing the inelastic epidermis, which released the web and flexion contractures. The fingers were separated by blunt dissection to the web base, and flexion contractures were addressed with transverse volar incisions extending to the finger sides (Figure 1c,d). After exposing the dermis, artificial dermis grafts were applied to cover the palm and fingers followed by application of polymyxin B‐soaked gauze to maintain a wet‐to‐dry environment and non‐adherent dressing. A boxing‐glove type dressing was used to maintain wrist dorsiflexion, metacarpophalangeal (MCP) and interphalangeal (IP) joint extension, and thumb abduction without Kirschner wires.

**FIGURE 1:**
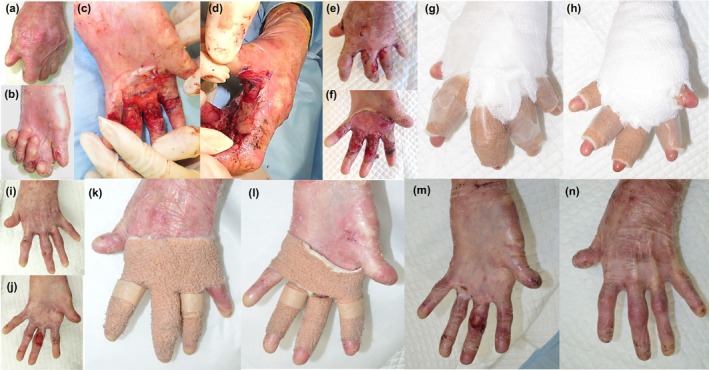
(a, b) Pseudosyndactyly of the left hand before surgery. (c, d) Flexion contractures were relieved by making transverse volar incisions at the level of flexion creases, which extended to the lateral sides of the fingers. (e–h) Postoperative day 7 and depressing. (i‐l) Nine weeks after surgery and depressing. (m, n) Three years and 8 months after surgery.

The first dressing change was on day 5 post‐surgery, with the removal of the silicon film from the artificial dermis grafts. Non‐adherent vaseline gauze (ADAPTIC^®^; 3M) was used to cover the hands, and an alginate coating dressing (KALTOSTAT^®^; Convatec) placed over it. Alginate dressing was also placed between the fingers to prevent web space obliteration and flexion contracture, with each finger fixed using a self‐adhesive bandage for traction (Figure 1e–h). Continued dressing changes maintained a good condition, and although complete epithelialization took over 9 weeks (Figure 1i–l), the patient was satisfied with the outcome and improved hand use in daily activities. Three years and 8 months postoperatively, there is no adhesion of the fingers, and the patient has had a favorable clinical course (Figure 1m,n).

Most RDEB patients opt for hand surgeries to regain function. Treatments such as allogeneic fibroblast injections and skin substitutes show benefits but are not curative, and recurrence is unavoidable.^2^ Box et al. reported that 71.4% of the survey respondents used pins during surgery and recommended wearing post‐operative hand orthoses to preserve surgical results. However, recurrence occurred in approximately 50% of cases after 1 year, requiring additional procedure.^3^ By utilizing postoperative compressive bandaging, the use of pins during surgery is avoided, which reduces the risk of infections such as osteomyelitis. Additionally, there is no need for specialized hand orthoses, and our patient has shown no need for reoperation even after a follow‐up period of 3 years and 8 months.

Our results show that surgical correction with postoperative compressive bandaging effectively improves pseudosyndactyly in RDEB patients. Continuous bandaging preserves range of motion and delays deformity recurrence, proving beneficial for RDEB patients.

## References

[1] Soro L , Bartus C , Purcell S . Recessive dystrophic epidermolysis bullosa: a review of disease pathogenesis and update on future therapies. J Clin Aesthet Dermatol. 2015;8:41–46. 26029334 PMC4445895

[2] Zhou X , Zhang Y , Zhao M , Jian Y , Huang J , Luo X , et al. Surgical management of hand deformities in patients with recessive dystrophic epidermolysis bullosa. J Plast Surg Hand Surg. 2020;54:33–39. 31502914 10.1080/2000656X.2019.1661846

[3] Box R , Bernardis C , Pleshkov A , Jessop N , Miller C , Skye J , et al. Hand surgery and hand therapy clinical practice guideline for epidermolysis bullosa. Orphanet J Rare Dis. 2022;17:438. 36522659 10.1186/s13023-022-02596-z PMC9756448

